# Flicker electroretinogram in newborn infants

**DOI:** 10.1007/s10633-022-09889-5

**Published:** 2022-10-06

**Authors:** James V. M. Hanson, Caroline Weber, Oliver A. Pfäffli, Dirk Bassler, Daphne L. McCulloch, Christina Gerth-Kahlert

**Affiliations:** 1 Department of Ophthalmology, University Hospital Zurich and University of Zurich, Zurich, Switzerland; 2 Department of Neonatology, Newborn Research, University Hospital Zurich and University of Zurich, Zurich, Switzerland; 3 Department of Ophthalmology, Cantonal Hospital Lucerne, Lucerne, Switzerland; 4 School of Optometry and Vision Science, University of Waterloo, Waterloo, Canada

**Keywords:** Flicker ERG, Newborn infant, Electroretinogram, Retinal development, Human neonate

## Abstract

**Purpose:**

To develop and validate a flicker electroretinogram (ERG) protocol in term-born neonates as a potential tool for assessing preterm infants at risk of developing retinopathy of prematurity.

**Methods:**

A custom flicker ERG protocol was developed for use with the hand-held RETeval® electrophysiology device. Feasibility of measuring flicker ERG through closed eyelids and without mydriasis was established in a pilot study enabling optimisation of the test protocol. Following this, healthy term-born neonates (gestational age 37–42 weeks) were recruited at the Neonatology clinic of the University Hospital Zurich. Flicker ERG recordings were performed using proprietary disposable skin electrodes during the first four days of life when the infants were sleeping. Flicker stimuli were presented at 28.3 Hz for a stimulus series at 3, 6, 12, 30, and 50 cd·s/m^2^, with two measurements at each stimulus level. Results were analysed offline. Flicker ERG peak times and amplitudes were derived from the averaged measurements per stimulus level for each subject.

**Results:**

28 term-born neonates were included in the analysis. All infants tolerated the testing procedure well. Flicker ERG recording was achieved in all subjects with reproducible flicker ERG waveforms for 30 and 50 cd·s/m^2^ stimuli. Reproducible ERGs were recorded in the majority of infants for the weaker stimuli (with detectable ERGs in 20/28, 25/28, and 27/28 at 3, 6, and 12 cd·s/m^2^, respectively). Flicker ERG amplitudes increased with increasing stimulus strength, with peak times concurrently decreasing slightly.

**Conclusion:**

Flicker ERG recording is feasible and reliably recorded in sleeping neonates through closed eyelids using skin electrodes and without mydriasis. Flicker ERG amplitude decreases for lower luminance flicker but remains detectable for 3 cd·s/m^2^ flicker in the majority of healthy term-born neonates. These data provide a basis to study retinal function in premature infants using this protocol.

## Introduction

In humans, the basic structures and connections of the adult retina are present, at least in the central retina, by the midpoint of gestation [1, 2]. Thereafter, the rapid growth and development of the retina is characterised by complex processes such as development of rods and cones, reorganisation and migration of photoreceptors with lengthening of inner and outer segments, increasing rhodopsin concentration, and migration of inner retinal neurons towards the periphery [1, 3]. This period of rapid retinal maturation extends beyond full term birth to the first few postnatal months and then proceeds more gradually into early childhood. The retina of a neonate born at term is approximately half the volume of the adult retina and shows significant immaturities in both structure and function [1].

Electroretinograms (ERGs), retinal responses to light stimuli, provide objective measures of retinal function [4]. With suitable stimuli (such as a stimulus–response series [5, 6]), ERGs can measure both the sensitivity and responsiveness of the retina and document the rapid maturation of retinal function. In healthy preterm infants, dark-adapted ERGs show an approximately tenfold increase in responsiveness and an improvement in sensitivity of 1.5 log units between preterm (31–36 weeks postmenstrual age, PMA) and 10 to 17 weeks post-term age [7–10]. Dark-adapted ERGs reflect the function of the rod system of the retina, while light-adapted ERGs and ERGs to rapid flicker stimulation reflect function of the cone system. Although cones develop earlier in gestation and mature less rapidly than rods, light-adapted ERGs also show marked increases in responsiveness, with 4.7- and 6.8-fold increases in amplitude for the light-adapted and flicker ERGs, respectively, between late preterm and early postnatal ages [7, 11].

In infants born prematurely, retinal maturation is vulnerable to risk factors such as hyper- and hypoxia, hypotension, infections, and suboptimal nutrition [12, 13]. These risks range from sub-clinical deficits of retinal function to retinopathy of prematurity (ROP) with sight-threatening complications [12, 14]. In severely preterm infants (< 30 weeks gestational age, GA, and/or birth weight < 1250 g), rigorous screening and treatment programmes are needed to detect and manage ROP. Current clinical ROP screening protocols recommend a systematic retinal examination by an experienced paediatric ophthalmologist or retinal specialist to assess retinal maturation and search for abnormalities of the retinal vasculature [15].

Current healthcare systems in developing, low- and middle-income countries support increasing survival rates for prematurely born infants. However, the infrastructure and resources required to adequately screen for, manage, and prevent ROP are often insufficient, which is reflected by a relatively high incidence of ROP [13, 14, 16]. In such areas, improvements in locally adapted screening and treatment programmes are urgent priorities to reduce the burden of lifelong visual impairment and blindness [17, 18]. Objective, low-cost screening methods capable of being performed by trained technicians, rather than experienced ophthalmologists, may offer promise in increasing coverage of ROP screening in such countries.

ROP emerges as preterm infants approach term age, coincident with the phase of rapid maturation of the retinal vasculature, the photoreceptors, and the ERG responses [19]. Deficits in dark-adapted ERG sensitivity reflect suboptimal nutrition in preterm infants [8, 20] and may precede the onset of ROP [9, 11]. Although light-adapted ERGs are less affected than dark-adapted ERGs in ROP [11], there is some evidence that flicker ERGs may also show dysfunction prior to ROP onset. Specifically, flicker ERG amplitudes were markedly diminished at 36 weeks PMA in two infants who later developed severe ROP compared with 47 infants who later showed no or only very mild ROP [21].

Standard ERG recording requires pupil dilation, a period of dark adaptation, an electrode in contact with the anterior eye, and an open eye, necessitating the use of an eyelid speculum in infants. Thus, in clinical settings, the potential benefit of monitoring retinal function, even in those at risk, may be outweighed by the demands of ERG testing. Recording only flicker ERGs could potentially shorten the test to the extent that it would gain feasibility as a functional screening test, as only a brief burst of stimuli without previous dark adaptation is required. ERG, including flicker ERG, has previously been suggested to be of value in the assessment of retinal vascular disorders such as diabetic retinopathy, as reviewed elsewhere [22, 23].

Recently, a portable hand-held device for measuring ERGs using full-field stimuli has been developed, along with proprietary skin electrodes that include recording, reference, and ground electrodes within a single adhesive strip (RETeval®, with SensorStrips®, LKC Technologies Inc., Gaithersburg MD, USA). Recent work employing this device has demonstrated the feasibility of recording flicker ERGs in a large paediatric cohort without mydriasis [24].

With these factors in mind, we aimed to develop and validate a rapid flicker ERG protocol for the non-invasive measurement of retinal function in newborn infants. In this manuscript, we firstly describe the process of developing and testing a suitable flicker ERG protocol, followed by the process of amending and validating the protocol on a larger cohort.

## Methods

We conducted a non-randomised cross-sectional study at the University Hospital Zurich as a collaboration between the Department of Neonatology, Maternity ward, and the Department of Ophthalmology. The study protocol was approved by the Ethics Committee of Zurich, Switzerland (BASEC 2019–01245), and was in accordance with the Declaration of Helsinki and the guidelines of Good Clinical Practice.

### Subjects

Newborn infants were recruited from the Maternity Ward of the University Hospital Zurich. Parents were informed about the project by the investigators, and written informed consent was provided by the parents of all participating babies. Healthy neonates with normal postnatal adaptation and with no apparent congenital abnormalities or suspected medical issues were eligible for the study.

Pilot data were gathered on five neonates to test the feasibility of minimally disruptive flicker ERG testing. Of these, two (both male) were term-born and examined on the 3rd and 4th days of life, female twins with a GA of 35 5/7 weeks were examined on the 5th day of life, and a prematurely born female with a GA of 26 4/7 weeks was examined 10 weeks 5 days after birth (corresponding to a PMA of 37 2/7 weeks). In addition, six adult female volunteers aged 22–59 years without ocular or general diseases or malformations also underwent flicker ERG testing to evaluate differences between infants and adult populations and to verify the recording protocol.

Following the pilot measurements, 28 term-born neonates (born after 37 weeks GA, [54%] female) were recruited between October 2019 and March 2020 and examined using the amended study protocol.

### Flicker ERG recording

Neonates were visited in their maternity room by the examiners. To minimise potential stress, the infants were swaddled in a blanket and, if necessary, given sucrose drops (for reassurance and analgesia [25]) or a pacifier/dummy. The infants received one to two sucrose drops (Algopedol® 24% sucrose) two minutes prior to examination after the consent of the parents was obtained.

During preliminary testing, some recordings were attempted when infants were awake to assess the impact of closed eyelids. These recordings were incomplete with an unacceptable number of movement artefacts. Thereafter, all recordings were performed only when the neonates were asleep. Each infant was tested on one occasion and in one eye only. The eye to be tested was dependent upon the posture and head position of each infant at the time of examination, with the most easily accessible eye being selected for examination. Examinations were performed in dim room illumination. Adult subjects were awake, with closed eyelids, during data acquisition. Iris colour (blue and brown) and lid pigmentation (light, medium, and heavy) were noted at the ERG-recording.

We used the RETeval Complete™ (LKC Technologies) hand-held ERG device running firmware version 2.9.4, with the manufacturers’ proprietary Sensor Strip™ disposable skin electrodes placed below the lower eyelid as specified. The skin was gently cleaned with small amounts of skin disinfectant and abrasive paste immediately before placing the electrodes, taking great care not to damage or excessively irritate the skin or to wake the sleeping babies. The flicker stimulus was delivered through closed eyelids with natural (i.e. undilated) pupils [26]. The eye position was verified by observing the position of the corneal bulge through the eyelid, prior to each step of recording to ensure that the eyes had not rotated upwards.

For the pilot study, we designed a custom test protocol which covered a range of stimulus levels from 1 to 50 cd·s/m^2^ with an approximately logarithmic progression (i.e. the stimulus levels were approximately equally spaced on a logarithmic scale), truncated at the maximum stimulus strength of the device: 1, 3, 10, 30, and 50 cd·s/m^2^. All stimuli were pulses of < 5 ms duration presented continuously with a flicker frequency of 28.3 Hz via the full-field stimulator held over the closed eyelid. The stimuli were produced by a combination of long (621 nm), medium (530 nm), and short (470 nm) wavelength LEDs balanced to achieve white flashes with CIE co-ordinates of x = 0.33, y = 0.33. To maximise the effect of the flicker stimuli, they were presented without background illumination. Sampling rate was 2 kHz. The ERG recordings were real-time processed in the frequency domain using the device-based proprietary software, which sets criteria based on the variance of the phase of the signal at the stimulus frequency [27]. Online artefact rejection was accomplished by a process of wavelet de-noising and calculation of the trimmed mean. Time-domain averaged waveforms were re-constructed over a temporal window of 1024 ms by adding the stimulus frequency and the first eight harmonics for display and assessment on the device after each measurement. Each recording lasted until the ERG met the phase criterion or for a maximum of 15 s.

As we were uncertain how the examination would be tolerated in neonates, we chose to begin the examination with 30 cd·s/m^2^ stimulation so that if it was not possible to complete the recording, we would have initially collected limited data to a stimulus which was expected to be above threshold. The stimuli were therefore applied in the order of 30, 1, 3, 10, and 50 cd·s/m^2^, followed by a repeated stimulation at 30 cd·s/m^2^. For each stimulus strength, two measurements were obtained.

### Analysis

Flicker ERGs were analysed from the tested eye of all infants and from the right eye only of adult subjects. After saving raw data files from the device onto a computer, proprietary software (RETeval® RFF Extractor, LKC Technologies) was used to extract the reconstructed voltage and timing data as .csv files which were then imported into Excel 2016 (Microsoft Corporation, Redwood WA, U.S.A.), for processing. All ERG waveforms were inspected for reproducibility (i.e. the two waveforms measured at each stimulus strength were visually compared) and averages calculated for each stimulus. Flicker ERG peak times and trough-to-peak amplitudes of the waveforms for each stimulus strength were extracted from the averaged data using custom-written code run in MATLAB (Mathworks, Natick MA, U.S.A.). The implicit time of the response peak following the first visible trough was analysed, following standard reporting methods for clinical flicker ERGs [4].

## Results

**Fig. 1:**
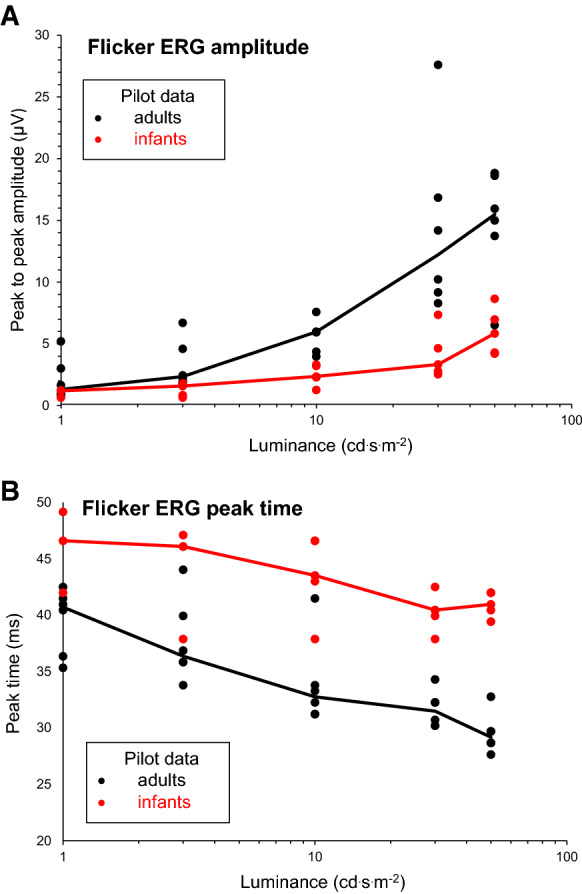
**A:** Amplitude of flicker ERG waveforms recorded without mydriasis through closed eyelids is shown for pilot data from adults (n = 6) and neonates (n = 5) tested at near term age. Dots show the individual data (average of two recordings); the lines are group median values. **B**: Implicit time to the first peak of flicker ERG waveforms recorded without mydriasis through closed eyelids is shown for pilot data from adults (n = 6) and neonates (n = 5). Dots show the individual data (average of two recordings); the lines are group median values

It was possible to record flicker ERG through closed eyelids using skin electrodes in all of the infants and adults examined in the pilot study. The procedure took less than 15 min per subject including preparation. All neonates tolerated the procedure well; however, the infants tended to become slightly restless toward the end of the recording, when the stronger stimuli (30 and 50 cd·s/m^2^) were used. As there were no apparent differences between the 30 cd·s/m^2^ ERG waveforms recorded at the beginning and the end of the pilot protocol, only those recorded at the beginning of the pilot protocol were analysed. The amplitude of the flicker ERGs recorded in adults were visibly larger, and the peaks earlier, than those recorded in infants. In two of the five infants in the pilot study, no response to 1 cd·s/m^2^ stimulation was detectable. The amplitudes of the recordings in adults and neonates approximately overlap when the stimulus is a log unit stronger in babies, as illustrated in Fig. 1. However, peak times are delayed in the newborn group even with a stronger stimulation, as listed in Table 1.

**Table 1:** Flicker ERGs from neonates and adults in the pilot study group

Flicker stimulus (cd·s/m^2^)	Amplitude (µV) Mean (SD) Median [range]		Peak Time (ms) Mean (SD) /Median [range]	
	n = 6 Adults	n = 5 Infants	n = 6 Adults	n = 5 Infants
1	2. (1.8)	1.0 (0.3)*	39.5 (2.9)	45.9 (3.6)*
	1.3 [4.5]	1.2 [0.6]*	40.7 [7.2]	46.6 [7.2]*
3	3.3 (2.0)	1.3 (0.5)	37.7 (3.7)	45.5 (4.6)
	2.4 [4.8]	1.6 [1.2]	36.4 [10.2]	46.1 [12.3]
10	5.7 (1.3)	2.5 (0.8)	33.9 (3.9)	43.5 (3.6)
	6.0 [4.8]	2.4 [2.1]	32.8 [10.2]	43.5 [8.0]
30	14.4 (7.3)	4.1 (2.0)	31.7 (1.6)	40.2 (1.6)
	12.2 [19.3]	3.3 [4.8]	31.5 [4.1]	40.5 [4.6]
50	14.8 (4.5)	6.0 (1.9)	29.5 (1.8)	41.0 (1.1)
	15.5 [12.3]	5.9 [4.4]	29.9 [5.1]	41.0 [2.6]

SD: standard deviation

^*^n = 3 infants for the 1 cd·s/m^2^ stimulus, as no reproducible waveforms were recorded in two of the five subjects

Based on the high success rate for the ERG protocol in infants, we concluded that the initial measurement at 30 cd·s/m^2^ was not necessary as a backup screening tool; rather, it was possible to perform a complete set of measurements in all infants. We decided to omit the weakest stimulus of 1 cd·s/m^2^ because it was not recordable in some of the neonates, and introduced an intermediate step of 6 cd·s/m^2^. Given that the infants only became restless with stronger stimuli, particularly the 50 cd·s/m^2^ stimulation, we amended the protocol to begin measurements with the weakest stimuli, progressing to the strongest stimuli. After consultation with the manufacturers of the RETeval®, we also shortened the maximum length of the recording for the 50 cd·s/m^2^ condition from 15 to five seconds to increase the tolerability of the protocol in newborns. Given the robust and reproducible waveforms generated by the 50 cd·s/m^2^ stimulus during the pilot study, we considered this an acceptable compromise to minimise potential disturbance to the neonates whilst maintaining adequate recording quality. Therefore, the amended test protocol included stimulus levels of 3, 6, 12, 30, and 50 cd·s/m^2^, in that order, delivered at a frequency of 28.3 Hz. In all other respects, the recording process was identical to that described in the previous Methods section.

Twenty-eight term-born neonates of median GA 39 weeks exactly were successfully examined using this revised recording protocol. As in the pilot study, the study eye was chosen based upon the position of the neonate whilst sleeping, with the right eye being tested in 39% (11/28) of infants. Demographics of the cohort are summarised in full in Table 2.

**Table 2:** Demographics of the main study cohort (n = 28 neonates)

Sex	Male: 13 (46%)
	Female: 15 (54%)
Postmenstrual age at birth, median in days [IQR]	273 [266–280]
Post-natal age at recording, median in days [IQR]	3 [3–4]
Eye measured	RE: 11/28 (39%)
	LE: 17/28 (61%)
Iris colour	Blue: 26 (93%)
	Brown: 2 (7%)
Lid pigmentation	Light to moderate: 26 (93%)
	Dark: 2 (7%)

IQR, interquartile range; LE, left eye; RE, right eye

**Fig. 2:**
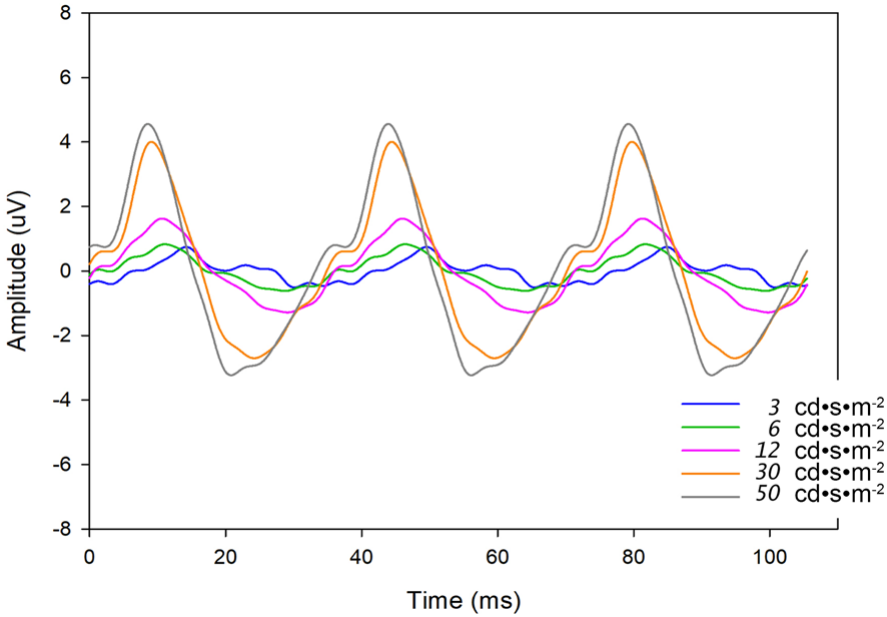
Representative 28.3 Hz flicker ERG waveforms for all five stimulus strengths recorded in a single neonate are superimposed. Each waveform is the average of two recordings

**Fig. 3:**
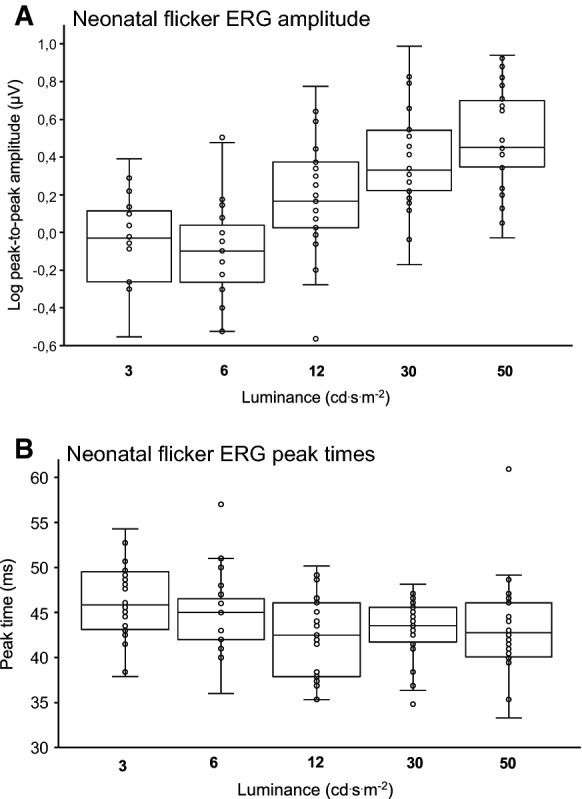
**A** Flicker ERG amplitudes are shown for 28 neonates born at term for 5 levels of flicker stimuli from 3 to 50 cd·s/m2. Horizontal bars are median values; boxes show interquartile range; capped lines show the range within which 95% of data points lie. **B**: Flicker ERG implicit time to the first peak are shown for 28 neonates born at term for 5 levels of flicker stimuli from 3 to 50 cd·s/m2. Horizontal bars are median values; boxes show interquartile range; capped lines show the range within which 95% of data points lie

Reproducible flicker ERG waveforms (defined as two individual responses with recognisable periodic peaks and troughs, exhibiting phase coherence) were detectable in the majority of the subjects at lower stimulus strengths: 20/28 (3 cd·s/m^2^), 25/28 (6 cd·s/m^2^), and 27/28 (12 cd·s/m^2^). Responses were reproducible in all infants at the higher stimulus strengths of 30 and 50 cd·s/m^2^. ERG amplitudes and peak times are provided in Table 3. Representative flicker ERG waveforms are illustrated in Fig. 2. Flicker ERG amplitudes increased with increasing stimulus strength as plotted in Fig. 3A and implicit times decreased with increasing stimulus strength as visible in Fig. 3B. Results for the two neonates with dark/heavily pigmented eyelids are provided next to the results of the remainder of the cohort in Table 4.

**Table 3:** Flicker ERG amplitudes and peak times in 28 term-born neonates

Stimulus Strength	Amplitude (µV)	Peak Time (ms)
(n ERGs*)	Mean (SD)	Mean (SD)
	Median [IQR]	Median [IQR]
3 cd·s/m^2^	1.0 (0.5)	46.2 (4.5)
(n = 20)	0.9 [0.6 – 1.3]	45.8 [43.1 – 50.0]
6 cd·s/m^2^	1.0 (0.7)	44.9 (4.3)
(n = 25)	0.8 [0.6 – 1.1]	45.0 [42.0 – 46.5]
12 cd·s/m^2^	1.9 (1.3)	42.4 (4.2)
(n = 27)	1.5 [1.1 – 2.4]	42.5 [37.9 – 46.1]
30 cd·s/m^2^	2.9 (2.0)	43.1 (3.5)
(n = 28)	2.1 [1.7 – 3.5]	43.5 [41.7 – 45.6]
50 cd·s/m^2^	3.7 (2.3)	43.2 (5.1)
(n = 28)	2.8 [2.2 – 5.0]	[40.1 – 46.1]

^*^The number of neonates in whom reproducible flicker ERGs were successfully recorded is given for each stimulus strength. Only one eye was tested for each neonate

IQR, interquartile range; SD, standard deviation

**Table 4:** Flicker ERG amplitudes and peak times in neonates with different skin pigmentation

Stimulus strength	Amplitude (µV)		Peak Time (ms)	
(cd·s/m^2^)	Light-medium skin* Mean (SD) Median [IQR]	Dark skin Mean (values)	Light-medium skin Mean (SD) Median [IQR]	Dark skin Mean (values)
3	1.0 (0.5)	NR	46.16 (4.45)	NR
	0.9 [0.6 – 1.3]		45.82 [43.14 – 49.54]	
	n = 20		n = 20	
6	1.0 (0.8)	1.1 (1.1, 1.1)	45.1 (4.3)	47.6 (45.1, 50.2)
	0.8 [0.6 – 1.2]		43.5 [42.5 – 46.6]	
	n = 23	n = 2	n = 23	n = 2
12	1.9 (1.3)	1.8 (1.2, 2.4)	42.2 (4.2)	45.6 (42.5, 48.6)
	1.5 [1.0 – 2.6]		42.0 [37.9 – 45.6]	
	n = 25	n = 2	n = 25	n = 2
30	3.0 (2.0)	2.6 (1.9, 3.3)	43.1 (3.6)	43.5 (43.0, 44.0)
	2.1 [1.6 – 3.5]		43.5 [41.3 – 45.6]	
	n = 26	n = 2	n = 26	n = 2
50	3.8 (2.4)	2.5 (2.2, 2.8)	43.3 (5.2)	41.0. (39.4. 42.5)
	2.9 [2.2 – 5.4]		43.0 [40.3 – 46.2]	
	n = 26	n = 2	n = 26	n = 2

*Caucasian infants with light-medium (fair) skin pigmentation (n = 26) and those with darkly pigmented eyelids (n = 2). In both infants with dark eyelid pigmentation, no responses were recorded to 3 cd·s/m^2^ stimulation. In the infants with light and medium skin pigmentation, the number of patients in whom flicker ERG could be successfully recorded is provided for each stimulus strength

IQR, interquartile range; NR, non-recordable; SD, standard deviation

Shapiro–Wilk testing (SigmaPlot 13, SyStat Software GmbH, Düsseldorf, Germany) indicated that both the ERG amplitudes and peak times were not normally distributed (data not shown), as is the case for many ERG parameters [4], and therefore parametric analyses (including linear regressions) were contraindicated. Instead, amplitude and peak time data for each of the stimuli were compared using Kruskal–Wallis one-way analysis of variance on ranks. This revealed that both amplitudes (*p* < 0.001) and peak times (*p* = 0.017) differed significantly according to stimulus strength.

## Discussion

In the present work, we have demonstrated the feasibility of flicker ERG recording in sleeping infants using skin electrodes and without mydriasis. The infants tolerated the preparation and recording extremely well. Increasing stimulus strength resulted in increased ERG amplitudes and, to a lesser extent, shorter ERG peak times. However, stimulation through closed eyelids to stimuli below 30 cd·s/m^2^ did not produce recordable responses in all infants. Although the natural pupil and closed eyelids attenuate the stimulus, and the eyelids likely act additionally as red filters, flicker ERGs are consistently recordable to stronger stimulation and may be useful in evaluation of retinal maturation and function.

During the course of the study, we observed that the examination was easiest to perform postprandial, when the sleeping infants were least likely to be restless. Comfortable swaddling and, when necessary, sucrose drops also helped to facilitate the examinations. We found that the most reliable examinations were obtained in these circumstances. In the cases where infants were slightly more active (e.g. in the period before feeding or when using brighter stimulation), repeated measures were sometimes necessary due to noisy recordings or visible artefacts. We propose that optimizing the timing and circumstances of the examination, which necessitates a degree of flexibility on the part of the examiner(s), is important to ensure reliable results.

Our infant cohort was predominantly pale skinned, with only two neonates (7%) having dark/heavily pigmented skin and eyelids. Whilst this was not unexpected, given the Swiss study site, we were nevertheless interested whether the test was still feasible in darker-skinned infants. Inspection of Table 4 confirms that this is the case: although it was not possible to record responses to the weakest stimulation in these infants, responses to all of the brighter stimuli were similar to those of the remainder of the cohort. Although the study was not powered to detect differences based on skin pigmentation, the presence of ERG waveforms in these two infants within the range of those for light skinned infants support the likelihood that it is also possible to use our flicker ERG protocol in babies of non-white ethnicities. In this regard, further studies with more ethnically diverse cohorts are necessary to characterise more precisely potential relationships between eyelid pigmentation and flicker ERGs recorded during sleep. We note that other authors have hypothesised that skin pigmentation may have a lesser effect on transmission of light through the eyelids than lid thickness [28].

The pilot study showed that our approach permits adequate ERG recording quality in both infants and adults. The very thin eyelids in our study population, neonates, ensure that responses can typically be evoked with similar stimuli to those used in adults even though the neonatal retinal sensitivity is lower. ERG amplitudes are typically two to three times smaller in neonates than in adults.

If validated in prematurely born infants at risk of developing ROP, such a minimally invasive flicker ERG protocol could be beneficial for understanding retinal function during early development in preterm infants. Advantages include ease of recording, with measurements which may be performed by trained technicians rather than ophthalmologists. The portability/hand-held nature of the RETeval™ relative to other ERG devices represent additional advantages. The main limitation of the strategy is that only the cones and cone pathways of the retina are assessed using flicker ERGs at the frequency employed here.

At the present time, the optimal stimulus parameters, accuracy, sensitivity, and specificity of flicker ERG screening for retinal function and any dysfunction associated with ROP are not known. One issue which may arise with very prematurely born infants is that the proprietary skin electrodes we used are physically too large for such small infants. We anticipate that specialised paediatric skin electrodes will be available in the near future. Further work should seek to validate and reproduce our results, to assess the feasibility and refine both testing protocols and analysis methods of our protocol in infants born both at term and prematurely. We plan to investigate the maturation of flicker ERGs in preterm infants and explore any association with risk of ROP and are currently in the process of extending our study to include infants born prematurely, including those at risk of ROP.

In summary, we have shown that it is feasible to record minimally invasive flicker ERG in sleeping term-born infants using skin electrodes and present preliminary normative data for healthy term-born infants. We would welcome verification and replication/extension of our results and will provide our testing protocol to other researchers upon request.

## Data Availability

The custom RETeval® protocol used in this study is freely available to all interested researchers by contacting LKC Technologies (support@lkc-com) or the corresponding author. Pseudonymised data may be provided upon reasonable request by qualified persons in writing to the corresponding author.

## Declarations

### Statement on the welfare of animals

No animals were used in this research.
